# Effects of Low-Dose and Very Low-Dose Ketamine among Patients with Major Depression: a Systematic Review and Meta-Analysis

**DOI:** 10.1093/ijnp/pyv124

**Published:** 2015-11-17

**Authors:** Ying Xu, Maree Hackett, Gregory Carter, Colleen Loo, Verònica Gálvez, Nick Glozier, Paul Glue, Kyle Lapidus, Alexander McGirr, Andrew A. Somogyi, Philip B. Mitchell, Anthony Rodgers

**Affiliations:** The George Institute for Global Health, The University of Sydney, Sydney, Australia (Drs Xu, Hackett, and Rodgers); Centre for Translational Neuroscience and Mental Health, University of Newcastle, Australia (Dr Carter); School of Psychiatry, University of New South Wales & Black Dog Institute, Sydney, Australia (Drs Loo, Gálvez, and Mitchell); Brain and Mind Research Institute, University of Sydney, Australia (Dr Glozier); Department of Psychiatry, University of Otago, New Zealand (Dr Glue); Departments of Psychiatry and Neurobiology, Stony Brook University, Stony Brook, NY (Dr Lapidus); Department of Psychiatry, University of British Colombia, Canada (Dr McGirr); Discipline of Pharmacology, Faculty of Health Sciences, The University of Adelaide, Adelaide, Australia (Dr Somogyi).

**Keywords:** Ketamine, major depression, meta-analysis

## Abstract

**Background:**

Several recent trials indicate low-dose ketamine produces rapid antidepressant effects. However, uncertainty remains in several areas: dose response, consistency across patient groups, effects on suicidality, and possible biases arising from crossover trials.

**Methods:**

A systematic search was conducted for relevant randomized trials in Medline, Embase, and PsycINFO databases up to August 2014. The primary endpoints were change in depression scale scores at days 1, 3 and 7, remission, response, suicidality, safety, and tolerability. Data were independently abstracted by 2 reviewers. Where possible, unpublished data were obtained on treatment effects in the first period of crossover trials.

**Results:**

Nine trials were identified, including 201 patients (52% female, mean age 46 years). Six trials assessed low-dose ketamine (0.5mg/kg i.v.) and 3 tested very low-dose ketamine (one trial assessed 50mg intra-nasal spray, another assessed 0.1–0.4mg/kg i.v., and another assessed 0.1–0.5mg/kg i.v., intramuscular, or s.c.). At day 3, the reduction in depression severity score was less marked in the very low-dose trials (P homogeneity <.05) and among bipolar patients. In analyses excluding the second period of crossover trials, response rates at day 7 were increased with ketamine (relative risk 3.4, 95% CI 1.6–7.1, *P=.*001), as were remission rates (relative risk 2.6, CI 1.2–5.7, *P*=.02). The absolute benefits were large, with day 7 remission rates of 24% vs 6% (*P=.*02). Seven trials provided unpublished data on suicidality item scores, which were reduced on days 1 and 3 (both *P*<.01) but not day 7.

**Conclusion:**

Low-dose ketamine appears more effective than very low dose. There is substantial heterogeneity in clinical response, with remission among one-fifth of patients at 1 week but most others having benefits that are less durable. Larger, longer term parallel group trials are needed to determine if efficacy can be extended and to further assess safety.

## Introduction

Aside from electro-convulsive therapy (ECT), there are no widely used treatments that provide large or rapid benefits for patients suffering from severe depression (Martinowich et al., 2013). This presents a major clinical challenge, especially for patients who have not responded fully to existing therapy or if symptoms include suicidality. In 2000 a small crossover trial suggested that a single subanaesthetic dose of ketamine could provide a large antidepressant benefit, starting within a few hours and lasting for at least several days (Berman et al., 2000). Ketamine appeared to improve specific depressive symptoms such as sadness, suicidality, and helplessness, rather than induce a nonspecific mood-elevating effect. Since then, several more trials have been published (Zarate et al., 2006; Diazgranados et al., 2010a; Zarate et al., 2012; Murrough et al., 2013; Sos et al., 2013), but existing reports and reviews (aan het Rot et al., 2010; Aan Het Rot et al., 2012; Katalinic et al., 2013; Brittner et al., 2014; Caddy et al., 2014; McGirr et al., 2014) do not easily allow a direct comparison of how treatment effects persist in the days following treatment, given differences in graphical and tabular reporting methods and outcome scales. Three very recently reported trials (Lai et al., 2014; Lapidus et al., 2014) (C. K. Loo, V. Galvez, E. O’Keefe, unpublished data) evaluated lower doses of ketamine than previously tested, providing an opportunity to compare treatment efficacy and tolerability of different doses. One other key issue is the extent of bias resulting from the use of a crossover design in all but one previous trial. Potential biases from crossover designs, such as carryover of treatment effect into the second treatment period, can be addressed by restricting analyses to the first treatment period but often requires unpublished data. We therefore conducted a systematic review of trials of ketamine in patients with treatment-resistant depression to evaluate antidepressant efficacy, suicidality, safety, and tolerability.

## Methods

### Types of Trials

We considered all relevant randomized trials (either crossover or parallel) in which ketamine was used specifically for the treatment of a Major Depressive Episode (DSM-IV diagnosis) and was compared with placebo, including active placebo. Eligible trials included participants with either unipolar or bipolar affective disorder. We excluded trials conducted in the context of ECT and surgery, but there were no restrictions on concomitant pharmacological or psychological treatments.

### Types of Interventions

We included any trial that attempted to evaluate a comparison between single administration of ketamine and placebo for the treatment of major depressive disorder. There was no restriction on ketamine regimen used (eg, dose or route).

### Types of Outcome Measures

The primary endpoint was change in depression severity scores from baseline on depression scales such as the Hamilton Depression Rating Scale (HAM-D; Hamilton, 1960) and/or the Montgomery Åsberg Depression Rating Scale [MADRS]) at the following times: day 1 (24 hours after first dose), day 3, and day 7. Other outcomes were: clinical remission (defined as HAM-D <7 or MADRS <10); clinical response (defined as ≥50% reduction in depression severity score); suicidality measures, including the suicidality item of HAM-D or MADRS; safety and tolerability as assessed by reported adverse events and dropouts.

### Search Methods for Identification of Trials

Reports were restricted to English language publications. Publications for this review were identified by searching Medline, Embase, and PsycINFO databases before August 2014 using the following search terms as free text or subject headings as appropriate for each database: (“ketamine” OR “NMDA receptor antagonist”) AND (“depression” OR “major depressive disorder” OR “bipolar depression” OR “depressive disorder” OR “dysthymic disorder” OR “treatment resistant depression”) AND (“ randomized controlled trial” OR “controlled clinical trial” OR “randomized” OR “placebo”) (full details available in supplementary Table 1 in the online data supplement). If additional studies cited in these articles met with these criteria, then they were also included.

### Selection of Studies

Two authors (A.R. and Y.X.) reviewed the search strategy and selected studies for inclusion in the review. In case of disagreement, M.H. arbitrated.

### Data Extraction and Analysis

When relevant publications were selected, 2 authors (A.R. and Y.X.) independently extracted information on year of publication, geographic location, sample size, loss to follow-up, mean age, study design, major inclusion and exclusion criteria, concomitant treatments, and outcome measures as well as safety and tolerability.

Potential sources of bias were identified for each trial using criteria recommended in the Cochrane Handbook (Higgins and Green, 2009). For random sequence generation, we categorized trials as “low risk” if random-number charts or coin tossing was used, as “unclear” if there were no details of sequence generation, and as “high risk” if sequence was generated by odd/even date of birth. For allocation concealment, we categorized trials as low risk if opaque or sealed envelopes were used, as unclear if there was no detailed information and as high risk if assignments could possibly be foreseen. For blinding, we categorized studies as low risk if blinding was ensured and unlikely to be broken, as unclear if an inactive placebo was used, and as high risk if outcome measurement was likely to be influenced by lack of blinding. For incomplete outcome data, we categorized trials as low risk if missing outcome data balanced in numbers and with similar reasons across intervention groups, as unclear if reports of drop- outs were insufficient, and as high risk if there were imbalances in numbers or reasons for missing data across intervention groups. For selective reporting, we categorized trials as low risk if there was a published protocol and the outcome measures listed in the protocol were reported, as unclear if protocol could not be found and as high risk if a described outcome measure in the protocol or methods was not reported in results. Assessment of period and carryover effects were listed under “other sources of bias.” We categorized crossover trials as high risk and parallel trials as low risk.

Depression severity score (HAM-D and/or MADRS) at each follow-up time point was calculated for each trial, for ketamine or placebo groups, and a placebo-corrected value for each time point was calculated. If not available in tabular form, HAM-D and/or MADRS results at different time points were estimated independently from Figureures in the articles by 2 authors. Placebo-corrected HAM-D scores were plotted separately for each trial with that outcome measure, and placebo-corrected MADRS scores for all trials with that outcome measure. In addition, we plotted a percentage reduction in depression severity score for all trials based on MADRS score where available and HAM-D for other trials. We conducted a meta-analysis of mean MADRS score at day 1, 3, and 7, with day of administration defined as day 0. These analyses were conducted initially to include the data from the single parallel trial and the first period of all crossover trials. We also compared the relative risk (RR) during the first period against the second period in crossover trials in order to examine the direction and magnitude of carryover effects. Meta-analysis of continuous outcomes was conducted using Stata13 (www.stata.com), Windows 7. Meta-analyses of response rates and clinical remission at days 1, 3, and 7 were conducted using Review Manager Version 5.3. software (RevMan, 2014) to estimate RR and number needed to treat (NNT). All meta-analyses were done using a random effects model. The I^2^ statistic was used to assess heterogeneity of trial results, and subgroup heterogeneity was tested using a chi-squared statistic (Higgins and Green, 2009). Data were available only on day 4 for one trial (Sos et al., 2013), and this was included in the day 3 category. Data were sought from the crossover trials for each treatment period separately.

## Results

### Search Results

**Figure 1.**
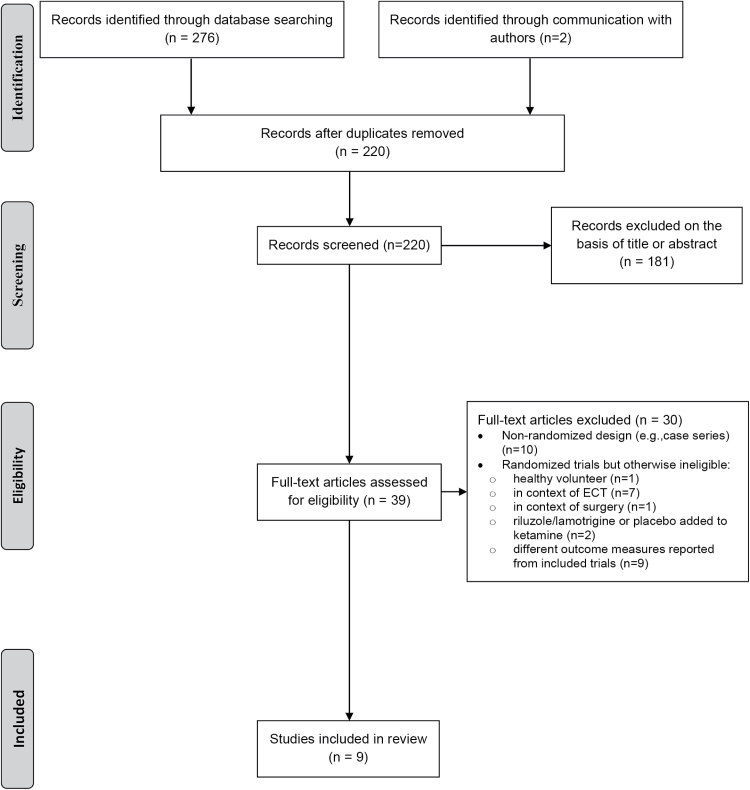
Flow diagram for systematic review.

The search results and study selection process are summarized in a PRISMA flowchart (Figure 1) showing the number of unique references identified by the search, the number of records excluded, and the number of full-text records retrieved. One placebo-controlled trial was not included because the sequence was not randomized (Valentine et al., 2011) and one randomized trial was not included because ketamine was compared with ECT (Ghasemi et al., 2014). One unpublished trial of the effects of intra-operative ketamine on depression was identified, but data were not available (M. Bastos, personal communication).

### Baseline Characteristics

**Figure 2.**
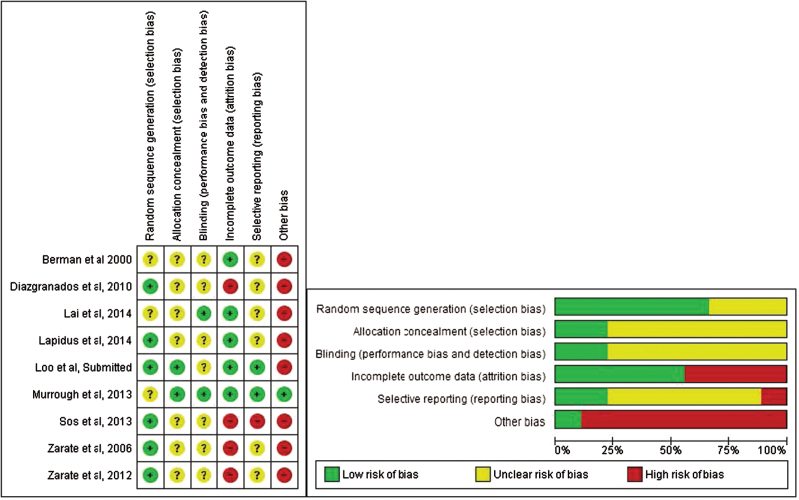
Potential sources of bias in included trials. See Methods for explanation of potential biases.

Nine randomized, placebo-controlled trials were identified, and a total of 201 patients (105 females and 96 males) were included (Table 1) (Berman et al., 2000; Zarate et al., 2006, 2012; Diazgranados et al., 2010a; Murrough et al., 2013; Sos et al., 2013; Lai et al., 2014; Lapidus et al., 2014) (C. K. Loo, V. Galvez, E. O’Keefe, unpublished data). Eight studies (Berman et al., 2000; Zarate et al., 2006, 2012; Diazgranados et al., 2010a; Sos et al., 2013; Lai et al., 2014; Lapidus et al., 2014) (C. K. Loo, V. Galvez, E. O’Keefe, unpublished data) were crossover trials with a 1- to 2-week washout period between treatments, and one was a parallel group design with 1 week of follow-up (Murrough et al., 2013). Two trials (Diazgranados et al., 2010a; Zarate et al., 2012) included only patients with bipolar disorder (BD), and 7 trials (Berman et al., 2000; Zarate et al., 2006; Murrough et al., 2013; Sos et al., 2013; Lai et al., 2014; Lapidus et al., 2014) (C. K. Loo, V. Galvez, E. O’Keefe, unpublished data) included patients with only unipolar depression (although one trial [Berman et al., 2000] included one patient with BD). All patients were treatment resistant, variously defined (Table 1). Patients with history of psychosis or recent substance use disorders were generally excluded. Patients in the 2 BD trials (Diazgranados et al., 2010a; Zarate et al., 2012) were drug free except for lithium or valproate. In the other 7 trials, patients were drug free in 2 (Berman et al., 2000; Zarate et al., 2006), allowed to take only a nonbenzodiazepine hypnotic in one (Murrough et al., 2013), and taking stable doses of other psychotropic medications in 4 trials (Sos et al., 2013; Lai et al., 2014; Lapidus et al., 2014) (C. K. Loo, V. Galvez, E. O’Keefe, unpublished data). Six trials (Berman et al., 2000; Zarate et al., 2006, 2012; Diazgranados et al., 2010a; Murrough et al., 2013; Sos et al., 2013) assessed a single 40-minute infusion of i.v. ketamine at a subanaesthetic low dose of 0.5mg/kg (defined here as low dose). Three trials tested lower doses (defined here as very low-dose trials): 50mg intranasally (as 10mg every 5 minutes), estimated to achieve plasma concentrations equivalent to about 0.3mg/kg i.v. (Lapidus et al., 2014); 0.1 to 0.4mg/kg i.v. (Lai et al., 2014), or 0.1 to 0.5mg/kg i.v., intramuscular, or s.c. in an ascending dose design (with placebo randomly inserted) (C. K. Loo, V. Galvez, E. O’Keefe, unpublished data). Seven trials (Berman et al., 2000; Zarate et al., 2006, 2012; Diazgranados et al., 2010a; Sos et al., 2013; Lai et al., 2014; Lapidus et al., 2014) used 0.9% saline as placebo and 2 used 0.045mg/kg (Murrough et al., 2013) or 0.01mg/kg (C. K. Loo, V. Galvez, E. O’Keefe, unpublished data) midazolam as an active placebo. Potential sources of bias are summarized in Figure 2.

**Table 1.** Characteristics of Previous Trials of One-Off Ketamine in Patients with Severe Mood Disorder

**Trial**	**Region,** **Country**	**Sample Size** ^*b*^ **(N)**	**Mean Age (SD),** **(y)**	**Male (N**)	**Design**	**Major Inclusion Criteria**	**Major Exclusion Criteria**	**Intervention**	**Control**	**Concomitant Treatments**	**Loss to Follow-Up**	**Outcome Measures†**
Berman et al., 2000	Connecticut	9	37 (10)	4	Crossover (washout ≥1 week)	Recurrent unipolar major depression (N=8); Bipolar disorder (N=1)	Recent alcohol or substance abuse; any other Axis I disorders	Ketamine i.v. 0.5mg/kg	Saline placebo i.v.	Nil and drug free 2 weeks prior	2 before last treatment (1 placebo, 1 ketamine), attended all previous follow-ups.	HAM-D-25, BDI, (VAS-high), (BPRS)
Zarate et al., 2006	Bethesda, Maryland	18	47 (11)	6	Crossover (washout 1 week)	Major depression disorder, DSM-IV; failed ≥2 antidepressant trials; HAMD-21 ≥ 18	Psychotic features; bipolar disorder; history of antidepressant- or substance- induced hypomania/mania	Ketamine i.v. 0.5mg/kg	Saline placebo i.v.	Nil and drug free 2 weeks prior	4 before last treatment (placebo), attended all previous follow-ups; 1 after first treatment (placebo), didn`t attend follow-up after Day 2.	HAM-D-21^*a*^, BDI, (BPRS), (YMRS), VAS-depression
Diazgranados et al., 2010a	Bethesda, Maryland	18	48 (13)	6	Crossover (washout 2 weeks)	Bipolar I or II depression, DSM-IV, failed antidepressant trial and mood stabilizer; MADRS ≥ 20; current major depressive episode ≥4 weeks	Psychotic features; substance abuse or dependence in last 3 months; unstable medical condition; serious risk of suicide; previous treatment with ketamine	Ketamine i.v. 0.5mg/kg	Saline placebo i.v.	Lithium/ valproate no other drugs during trial nor in prior 2 weeks (5 for fluoxetine); no psychotherapy	3 after first treatment (2 ketamine, 1 placebo), didn`t finish follow-ups. 2 after last treatment (both ketamine), didn`t finish follow-ups.	MADRS^*a*^, HAM-D-17, BDI, VAS-depression, VAS-anxiety, HAM-A, (BPRS), (YMRS)
Zarate et al., 2012	Bethesda, Maryland	15	47 (10)	7	Crossover (washout 2 weeks)	Bipolar I or II depression, DSM-IV, failed antidepressant trial and mood stabilizer; MADRS ≥ 20; current major depressive episode ≥4 weeks	Psychotic features; unstable medical condition; substance abuse or dependence in last 3 months; previous treatment with ketamine	Ketamine i.v. 0.5mg/kg	Saline placebo i.v.	Lithium/ valproate no other drugs during trial nor in prior 2 weeks (5 for fluoxetine); no psychotherapy	4 after first treatment (3 ketamine, 1 placebo), didn`t finish follow-ups.	MADRS^*a*^ HAM-D-17, BDI, VAS-depression, VAS-anxiety, HAM-A, (BPRS), (CADSS), (YMRS)
Murrough et al., 2013	Houston, Texas and New York City	72	47 (13) Ketamine 43 (12) Midazolam	35	Parallel	Major depressive disorder, DSM-IV; failed ≥3 antidepressant trials; ≥1 previous major depressive episode or lasting ≥2 years; ≥32 IDS-C	Psychotic illness or bipolar disorder; alcohol/substance abuse in the previous 2 years; unstable medical illness; serious and imminent suicidal or homicidal risk; <27 on MMSE	Ketamine i.v. 0.5mg/kg (N=47)	Midazolam i.v. 0.045mg/ kg (N=25)	Only prn nonbenzodiazepine hypnotic; drug free 1 week before (4 weeks for fluoxetine); no psychotherapy	2 from ketamine arm: both after 24-hour evaluation; 3 from placebo arm: all after 24-hour evaluation	MADRS^*a*^, QIDS-SR, CGI, (CADSS), (BPRS), (YMRS);
Sos et al., 2013	Prague, Czech Republic	30	42 (15) Ketamine first 45 (11) placebo first	15	Crossover (washout 1 week)	Major depressive disorder, DSM- IV; score of ≥20 on MADRS at baseline	Suicide risk, any other Axis I or II disorders; unstable medical illness or neurological disorder; psychotic symptom/ disorder in 1^st^/2^nd^ degree relatives; electroconvulsive therapy within 3 months	Ketamine i.v. Loading: 0.27mg/kg Maintenance: 0.27mg/kg	Saline placebo i.v.	Remained on antidepressant medications and dosages maintained throughout the duration of the study	1 before last treatment (ketamine), attended all previous follow-ups. 2 after first treatment (ketamine), didn`t finish follow-ups. 5 after last treatment (3 ketamine, 2 placebo), didn`t finish follow-ups.	MADRS^*a*^ (BPRS)
Lapidus et al., 2014	New York City, USA	20	48 (13)	10	Crossover (washout ≥1 week)	Major depressive disorder, DSM-IV; failed ≥1 trial of adequate dose and duration of antidepressant; IDS-C ≥ 30	Any other Axis I disorders; suicide risk; substance abuse or dependence in last 6 months; psychotic disorder; bipolar disorder; developmental disorder; previous abuse/ dependence on ketamine	Ketamine intranasal 50 mg	Saline placebo intranasal	Stable doses of psychotropic medication, including antidepressant treatment	2 before first treatment (1 ketamine, 1 placebo).	MADRS^*a*^, QIDS-SR, HAM-A, (BPRS), (CADSS), (YMRS), (SAFTEE)
Lai et al., 2014	Sydney, Australia	4	51 (17)	2	Crossover (washout 1 week) 4 dosages of ketamine	Major depressive episode ≥4 weeks, DSM-IV; MADRS ≥ 20; failed ≥ 1 trial of antidepressant during current episode	Comorbid Axis I or II disorders (aside from one subject with phobic disorder);	Ketamine i.v. 0.1, 0.2, 0.3, 0.4mg/Kg	Saline placebo i.v.	Remain on stable doses of psychotropic medications; no changes in medication dosage and no ECT 4 weeks prior to trial entry	1 after placebo, 0.1, and 0.2mg/kg (attended all previous follow-ups); 1 after placebo, 0.1, 0.2 and 0.3mg/kg (attended all previous follow-ups).	MADRS^*a*^, CGI, QIDS-SR, (BPRS), (YMRS), (CADSS), (SAFTEE).
C. K. Loo, V. Galvez, E. O’Keefe, unpublished data	Sydney Australia	15	49 (11)	11	Crossover (washout 1 week) 5 dosages of ketamine (i.v., N=4); (IM, N=5); (SC, N=6)	Major Depressive Disorder, depression episode of duration ≥ 4 weeks, DSM IV; MADRS ≥ 20; failed to ≥1 trial of antidepressant during current episode	Schizophrenia, rapid cycling bipolar disorder or any current psychotic symptoms	Ketamine i.v., IM or SC; 0.1, 0.2, 0.3, 0.4, 0.5mg/kg	Midazolam i.v., SC or IM 0.01mg/kg	Remain on stable doses of psychotropic medications; no changes in medication dosage and no ECT 4 weeks prior to trial entry	1 after placebo, 0.1, and 0.2mg/kg i.v.; 1 after placebo, 0.1, and 0.2mg/kg IM; 1 after placebo, 0.1, 0.2, and 0.3mg/kg IM; 1 after placebo, 0.1, 0.2, 0.3 and 0.4mg/kg IM; 1 after placebo, 0.1 and 0.2mg/kg SC; 1 after placebo SC;	MADRS^*a*^, (BPRS), (YMRS - item 1), (CADSS), (SAFTEE)
Total/ average		201	46	96(48%)	129 (64%) patients in crossover			Ketamine:145 (82%) i.v.; 20 (11%) intranasal; 5 (3%) intramuscular; 6 (3%) s.c.	Saline:94 (61%) i.v.; 20 (13%) intranasal; Midazolam:29 (19%) i.v.; 5 (3%) intramuscular; 6 (4%) s.c.	27 (13%) no concomitant medications;33 (16%) on Lithium/valproate; 72 (36%) only nonbenzodiazepine hypnotic; 69 (34%) on other psychotropic medications	40	

Abbreviations: MMSE: Mini-Mental State Examination; IDS-C, the Inventory of Depressive Symptomatology—Clinician Rated. †HAM-D, Hamilton Depression Rating Scale scores; BDI, Beck Depression Inventory; VAS(-high), Visual Analog Scales score (for intoxication “high”); BPRS, Brief Psychiatric Rating Scale;YMRS, Young Mania Rating Scale; MADRS, Montgomery-Åsberg Depression Rating Scale; HAM-A, Hamilton Anxiety Rating Scale; CADSS, Clinician Administered Dissociative States Scale; QIDS-SR,Quick Inventory of Depressive Symptomatology-Self Report; CGI, Clinical Global Impression; SAFTEE, Systematic Assessment for Treatment Emergent Effects.Measures in parentheses indicate the safety and tolerability measures.

^*a*^Primary outcome measures.

^*b*^Sample size was identified at the time of randomization, with an exception for Murrough et al., 2013. There were 73 patients at the time of randomization and 72 remained after allocation. One patient in the ketamine group did not receive intervention. Characteristics (eg, age and sex) were reported based on the 72 patients.

### Effects on Depression Severity Scores over Time

**Figure 3.**
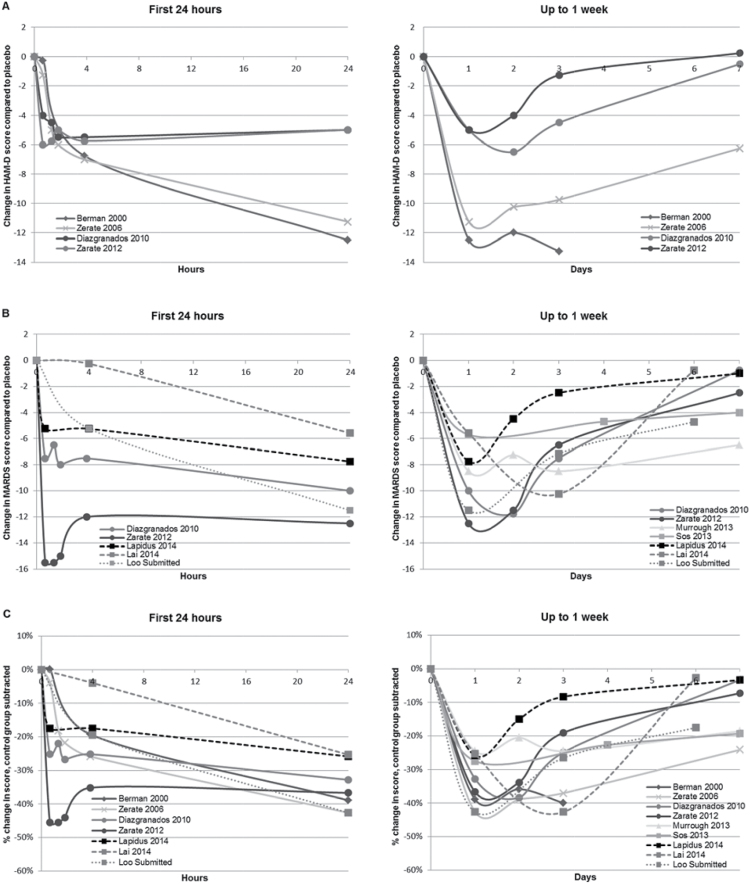
Reductions in depression severity scores following single-dose ketamine in patients with major depression. (A) Placebo-corrected changes in Hamilton Depression Rating Sclae Scores (HAM-D). (B) Placebo-corrected changes in Montgomery-Åsberg Depression Rating Scale (MADRS). (C) Placebo-corrected percentage changes in HAM-D/MADRS. Data are placebo-corrected, and axes redrawn on a linear scale to avoid a visual misrepresentation of how the treatment effect evolves over time. Lines with circular markers represent trials among patients with bipolar disorder depression. Other trials were among patients with unipolar depression. Lines with square markers represent patients remained on pretrial antidepressant treatment. Solid lines represent low dose (i.v. ketamine 0.5mg/kg). Dashed lines represent very low dose (intranasal ketamine 50mg or i.v. 0.3mg/kg; Lai et al. and Loo et al. used an ascending dose design and the data for 0.3mg/kg are shown).

**Figure 4.**
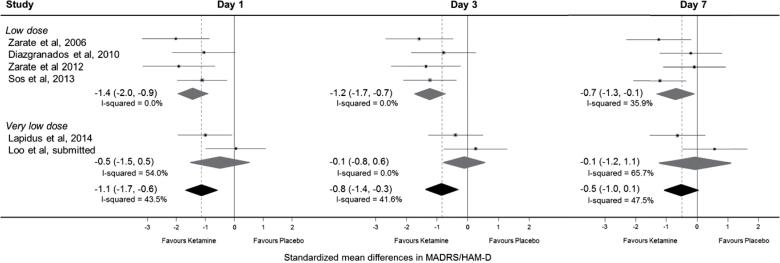
Difference in standardized mean mood score on days 1, 3, and 7 for crossover trials, first period only. Data are shown only for the first period in crossover trials. Hamilton Depression Rating Sclae Scores (HAM-D) was reported by Zarate et al., 2006 with the remainder reporting Montgomery-Åsberg Depression Rating Scale (MADRS). 95% CIs of treatment effects are represented by horizontal lines for individual trials, by grey diamonds for trial subgroups, and by black diamonds for all trials. A vertical dashed line goes through the overall pooled result for all trials, with a result to the left of the vertical line indicating a reduction in mood score in the ketamine group.

Effects on depression severity over time are presented in Figure 3, with data redrawn from original trials and placed on a uniform linear scale, and shown in Figure 4 with data from the single parallel trial and from the first period of crossover trials. A large reduction in depression severity was evident within 4 hours in all but one small trial of very low-dose ketamine, and treatment effects were largest at day 1. In the trials of very low-dose ketamine (Lai et al., 2014; Lapidus et al., 2014) (C. K. Loo, V. Galvez, E. O’Keefe, unpublished data), the reduction in overall severity appeared smaller and shorter lived. A test for heterogeneity indicated a significant difference in treatment effects at day 3 for low-dose compared with very low-dose trials (*P=.*02). In the 4 trials conducted with ketamine 0.5mg/kg i.v. for patients with unipolar depression (Berman et al., 2000; Zarate et al., 2006; Murrough et al., 2013; Sos et al., 2013), the large reduction in average mood score at 24 hours remained evident, though moderately attenuated, up to day 7. In the 2 trials among patients with BD, the treatment effect largely dissipated by days 4 to 7 (Diazgranados et al., 2010a; Zarate et al., 2012). A smaller treatment effect in BD patients compared with unipolar patients on the HAM-D was evident by 24 hours. There was no evidence of greater benefit in trials that aimed to attain high peak concentrations, either with an i.v. push during a few minutes (Lai et al., 2014) or an initial loading dose of 0.27mg/kg during the first 10 minutes (Sos et al., 2013).

### Effects on Response and Remission Rates over Time

**Figure 5.**
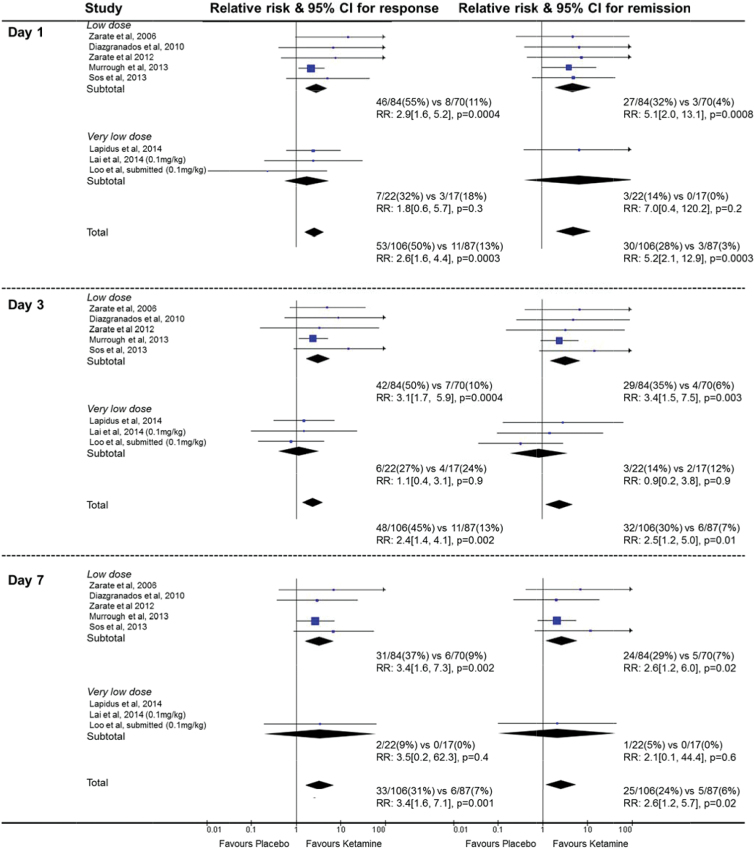
Effects of single-dose ketamine on response and remission rates at days 1, 3, and 7. Data included from the parallel trial (Murrough et al.) and from the first period of crossover trials. 95% CIs of treatment effects are represented by horizontal lines for individual trials and by diamonds for subgroups or all trials. Results on the right side of the vertical line indicated benifit in the ketamine group.

Analyses of treatment effects on response and remission, including only the single parallel group trial and the first treatment period of crossover trials, are summarized in Figure 5. At day 1, there was a large treatment effect, with 50% of those in the ketamine group compared with 13% in the placebo group meeting criteria for response (RR 2.6, 95% CI 1.6 to 4.4, *P=.*0003; NNT 2.9, 1.9–7.1). There was a similarly large effect on remission of symptoms at day 1 (RR 5.2, 95% CI 2.1–12.9, *P=.*0003; NNT 4.5, 3.1–8.3) among those receiving ketamine. At day 7, response rates were substantially increased in the ketamine group and to a larger degree (RR 3.4, 95% CI 1.6 to 7.1, *P=.*001; NNT 6.3, 3.4–25). Remission rates were still substantially increased in the ketamine group, although to a lesser degree (RR 2.6, 95% CI 1.2–5.7, *P=.*02; NNT 9.1, 5–50). While the pooled results of 3 trials of very low-dose ketamine showed no significant effect on response or remission on days 1, 3, or 7 and the treatment effect appeared smaller compared to low-dose ketamine, formal subgroup heterogeneity tests were not significant (*P=.*09 for response and *P=.*12 for remission on day 3).

One of the major potential biases in crossover trials is dropout after the first treatment, and this occurred in 12/46 patients who received ketamine initially compared with 5/55 who received placebo initially in 5 trials (Zarate et al., 2006, 2012; Diazgranados et al., 2010a; Sos et al., 2013; Lapidus et al., 2014). Dropout was for improved mood for 4 patients in the ketamine group and 2 in the placebo group. In terms of treatment effects in the second period of the crossover trials, overall 0/34 patients responded at day 1 who received placebo as their second treatment compared with 22/50 patients responding at day 1 after receiving ketamine as their second treatment. Remission was achieved in 0/34 patients at day 1 who received placebo as their second treatment compared with 8/50 patients after receiving ketamine as their second treatment. Thus, it did appear that carryover effects were not marked, although this cannot be assessed reliably since fewer patients in the ketamine groups proceeded to the second period.

### Effects on Suicidality

**Figure 6.**
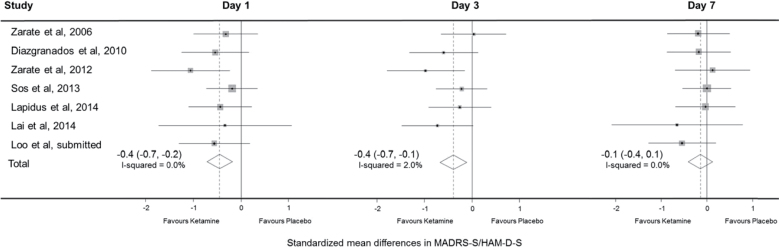
Standardized mean differences in suicide item scores at days 1, 3, and 7. Suicide item score from Hamilton Depression Rating Sclae Scores (HAM-D) provided by Zarate et al., 2006, and from Montgomery-Åsberg Depression Rating Scale (MADRS) for other trials. 95% CIls of treatment effects are represented by horizontal lines for individual trials and by diamonds for all trials. A vertical dashed line goes through the overall pooled result for all trials, with a result to the left of the vertical line indicating a reduction in mood suicide item score in the ketamine group.

Measures of suicidality were published in 4 trials (Berman et al., 2000; Zarate et al., 2006, 2012; Murrough et al., 2013) and these are summarized in Table 2. Each trial reported a significant reduction in suicidality assessed using different measures. Seven trials provided unpublished data on the suicide item component of a depression scale, 1 for HAM-D (Zarate et al., 2006), and 6 for MADRS (Diazgranados et al., 2010a; Zarate et al., 2012; Sos et al., 2013; Lai et al., 2014; Lapidus et al., 2014) (C. K. Loo, V. Galvez, E. O’Keefe, unpublished data). Overall, reported suicidality scores were low at baseline, with an average of 1.6 on the MADRS suicidality item (which ranges from 0 to 6) for trials that reported this outcome, as shown in Table 3. Nonetheless, a significant reduction in suicidality severity score was observed for the ketamine group at days 1 and 3, as seen in Figure 6.

**Table 2.** Published Measures of Suicidality

Trial	Measure Used	Outcome Reported in Publication
Berman et al., 2000	HAM-D suicidality item	While undergoing active treatment, significant decreases were observed for suicidality (*P* < .02). Control treatment was not associated with significant improvement in any of the HAM-D items.
Zarate et al., 2006	HAM-D suicidality item	Significant main effect for drug
Zarate et al., 2012	Changes in suicide item scores on the MADRS, HAM-D, and BDI	Within 40min, suicidal ideation signiﬁcantly improved in subjects receiving ketamine compared with placebo (Cohen’s d 0.98, 95% CI 0.64 –1.33); this improvement remained signiﬁcant through day 3. Reductions in suicide item scores for each of the MADRS, HAM-D, and BDI using linear mixed models (each *P* <.001)
Murrough et al., 2013	Composite index of explicit suicidal ideation (Beck Scale for Suicidal Ideation, MADRS suicide item, Quick Inventory of Depressive Symptoms suicide item)	Fifty-three percent of ketamine-treated patients scored 0 on all 3 explicit suicide measures at 24h compared with 24% of the midazolam group (χ = 4.6; *P* = .03)

Abbreviations: BDI, Beck Depression Inventory; HAM-D, Hamilton Depression Rating Scale; MADRS, Montgomery Åsberg Rating Scale.

**Table 3.** Unpublished Data on Suicidality Scores at Baseline

**Trial**	**Suicidality Item Subscale Used**	**Ketamine**		**Placebo**	
		**Mean**	**SD**	**Mean**	**SD**
Zarate et al., 2006	HAM-D, item 4*	1.9	1.1	1.2	1.1
Diazgranados et al., 2010a	MADRS, item 10**	2.0	1.9	2.4	1.9
Zarate et al., 2012	MADRS, item 10**	2.3	1.6	2.5	1.5
Sos et al., 2013	MADRS, item 10**	0.4	0.7	0.4	0.7
Lapidus et al., 2014	MADRS, item 10**	1.7	1.4	1.7	1.7
Lai et al., 2014	MADRS, item 10**	1.8	1.0	1.8	1.0
Loo et al., unpublished	MADRS, item 10**	1.9	1.2	2.3	1.0

Abbreviations: MADRS, Montgomery Åsberg Rating Scale.

*Ranges from 0 to 4; **ranges from 0 to 6.

### Tolerability and Side Effects

Measures of tolerability and side effects were summarized in Table 4. Low-dose ketamine at 0.5mg/kg, infused over 40 minutes, was generally well tolerated, with transient, mild-to-moderate dissociative symptoms and blood pressure and/or heart rate increases in a minority of patients. For example, in the largest trial, 17% of patients had significant dissociative symptoms immediately after ketamine infusion, but these all resolved within 2 hours and no severe psychotic symptoms (paranoia, hallucinations, or delusions) occurred in either arm (Murrough et al., 2013). The characteristic symptomatic side effects (eg, confusion, blurred vision) and the increases in heart rate and blood pressure resolved within 4 hours of administration in all trials. These side effects were less marked with very low-dose ketamine delivered intranasally for 20 minutes (Lapidus et al., 2014) but were more marked with very low-dose ketamine given as an i.v. push for a few minutes (Lai et al., 2014). Among trials that reported a full listing of adverse events in both groups, there was no excess in the ketamine vs placebo after administration day (Murrough et al., 2013; Lai et al., 2014; Lapidus et al., 2014) (C. K. Loo, V. Galvez, E. O’Keefe, unpublished data).

**Table 4.** Measures of Safety and Tolerability in Included Trials

**Trial**	**VAS-High**	**BPRS**	**CADSS**	**YMRS**	**SAEs**
Berman et al., 2000	Ketamine produced markedly greater scores. Scores are significantly different between two groups till 40min and return to baseline by 110min.	Ketamine produced significantly greater scores, especially the positive symptoms. Scores are significantly different between 2 groups until 40min and return to baseline by 120min.	Not mentioned	Not mentioned	Not mentioned
Zerate et al., 2006	Not mentioned	The positive symptoms subscale scores were worse for participants receiving ketamine than those receiving placebo only at 40min.	Not mentioned	Worse for participants receiving ketamine than placebo at 40 minutes, but they were significantly better from days 1 to 2.	Nil
Diazgranados et al., 2010a	Not mentioned	Ketamine and placebo differed only at 40min, and this difference was due to a small, nonsignificant decrease with placebo and an even smaller increase with ketamine.	A ketamine/placebo difference was seen at 40min only (large increase on ketamine).	Patients receiving ketamine had higher scores at 40min but significantly lower scores at days 2 and 14. Compared with baseline, there was no significant change in manic symptoms in patients receiving placebo.	One patient developed manic symptoms in the ketamine group that resolved within 80min and an affective switch occurred for one patient in the placebo group.
Zarate et al., 2012	Not mentioned	No significant drug effect or interaction.	Higher values in patients receiving ketamine only at 40min.	No significant drug effect or interaction.	Nil
Murrough et al., 2013	Not mentioned	The positive symptoms subscale scores for ketamine patients beyond the 40min ranged from 4.02 to 4.04.	At 40min, the average score for the ketamine group was 14.7 (10.6–18.8), and 2.28 (0.0–4.8) for the midazolam group. Average scores for the ketamine group beyond 40min ranged between 0.065 and 0.533.	The first item of the YMRS at 40min was 0.6 for ketamine and 0.12 for midazolam group.	Patient 1: BP=73/40 (1min) HR <30 bpm (30sec), spontaneous recovery; Patient 2: Suicide attempt while tapering off of psychotropic medication, patient was hospitalized .
Lapidus et al., 2014	Not mentioned	No relationship between ketamine associated changes in dissociative or psychotomimetic symptoms and antidepressant response was found	No relationship between ketamine associated changes in dissociative or psychotomimetic symptoms and antidepressant response was found	Measured, but not reported	Nil
Lai et al., 2014	Not mentioned	Clear dose–response relationship for psychotomimetic symptoms occurring within 40min. Scores returned to pre- treatment levels within 4h for all subjects.	Clear dose–response relationship for psychotomimetic symptoms occurring within 40min. Scores returned to pre-treatment levels within 4h for all subjects.	Not mentioned	During 0.4mg/kg dosage Two patients: tachycardia (150 bpm); One patient: BP increase (140/80 to 195/105). Spontaneous recovery within 15 min
C. K. Loo, V. Galvez, E. O’Keefe, unpublished data	Not mentioned	No evidence of treatment emergent mania, at any time point, across routes of administration and doses	Dose-response relationship between psychotomimetic effects and ketamine treatment for all routes, with higher peak scores in the i.v. group. Scores resolved without intervention by 40 minutes post- injection for all routes.	No evidence of treatment emergent mania, at any time point, across routes of administration and doses	Across groups, MAP elevations did not exceed >20% from baseline, except for 4 patients^a^ (i.v., N=2; IM, N=2). These effects resolved by 30min without intervention. Increases in heart rate did not exceed 120% of baseline, except in three participants (N=1, i.v.; N=1 intramuscular; N=1, S.C.).

Abbreviations: AE, adverse event; BPRS, Brief Psychiatric Rating Scale; CADSS, Clinician Administered Dissociative States Scale; CGI, Clinical Global Impression; MAP, mean arterial pressure; QIDS-SR, Quick Inventory of Depressive Symptomatology-Self Report; SAE, serious adverse event; SAFTEE, Systematic Assessment for Treatment Emergent Effects; VAS-high, Visual Analog Scales score for intoxication “high”; YMRS, Young Mania Rating Scale.

^a^In the i.v. group, 1 participant experienced a 25% increase 1 hour posttreatment (0.1 mg/kg) and a 24% MAP increase 5 min posttreatment (0.2–0.5 mg/kg). A second participant experienced a 44% MAP increase 10 min posttreatment (0.1 mg/kg). In the IM group, 1 participant experienced a 30% MAP increase 10 min posttreatment (0.4 mg/kg) and a second experienced a 39% MAP increase 5 min posttreatment (0.3 mg/kg).

Eleven events were reported as serious adverse events in the ketamine group: hypotension and bradycardia occurred in 1 person, which resolved in <1 minute and was considered to be due to a vaso-vagal episode (Murrough et al., 2013); suicide attempt occurred in 1 patient while tapering off of psychotropic medication (Murrough et al., 2013); tachycardia (>150 bpm) occurred in 2 patients (Lai et al., 2014); and mean arterial pressure elevations >20% from baseline in 5 patients (Lai et al., 2014) (C. K. Loo, V. Galvez, E. O’Keefe, unpublished data). Also, while not reported as serious adverse events, one ketamine-treated patient developed manic symptoms that resolved within 80 minutes, and an affective switch occurred for one patient in the placebo group (Diazgranados et al., 2010a). All the hemodynamic side effects resolved within a few hours with no lasting effects. Thus, it could be summarized that 3 major psychiatric events (suicide attempt before treatment, transient manic symptoms, and affective switch) were reported in the studies, of which only one was potentially attributable to ketamine (transient manic symptoms). No major medical events occurred in the trials.

Diverse measures were used to test possible psychotomimetic, dissociative, or mood elevation symptoms, including Visual Analogue Scale score for intoxication “high” (Aitken, 1969), Brief Psychiatric Rating Scale (Overall and Gorham, 1962), Young Mania Rating Scale (Young et al., 1978), and Clinician Administered Dissociative States Scale (Bremner et al., 1998). The results showed that Visual Analogue Scale score for intoxication “high” scores returned to baseline within 110 minutes of the infusion (Berman et al., 2000). In no case did euphoria, derealization, or depersonalization persist beyond 110 minutes (Zarate et al., 2006).

Continuation into the second phase of the crossover trial was also assessed as a proxy of tolerability, and most dropouts were reported as being due to changes in mood rather than adverse events. In 5 crossover trials with first-phase data available (Zarate et al., 2006, 2012; Diazgranados et al., 2010a; Sos et al., 2013; Lapidus et al., 2014), 12 of 46 patients did not proceed to placebo treatment after receiving ketamine first. For 4 patients, this was due to improved mood (Zarate et al., 2006), for 4 it was due to worsening mood (Diazgranados et al., 2010a; Sos et al., 2013), and other reasons were involved for an additional 4 (Zarate et al., 2012; Lapidus et al., 2014).

## Discussion

### Summary of Findings

This systematic review of trials of single-dose ketamine compared with placebo for treatment-resistant patients in a major depressive episode confirms a large reduction in depression severity and suicidality that is apparent within 4 hours after ketamine administration. The data suggest benefits are smaller and shorter lived with very low-dose ketamine. There is also a suggestion of short-lived effects in patients with BD. There is substantial heterogeneity in clinical response, with benefits lasting <1 week for most patients, but approximately one-fifth of patients remaining in remission at 1 week.

### Limitations

This systematic review has a number of methodological limitations. Most important is the small sample size (average of only 23 patients per trial) and 201 patients in total. The use of a crossover design in all but one trial is a potential issue. While a crossover design improves study power to partly mitigate problems associated with small sample size, it can have limitations for mood disorder trials. Crossover designs are most reliable when assessing a fully reversible treatment, with outcomes that return to baseline levels after a suitable washout period (ie, short-lived interventions that do not affect the natural history of a stable illness). For treatments that do not have these criteria, one can observe period effects (ie, the treatment effect in the first period is systematically different from that in the second period) and/or carryover effects (ie, the treatment effect in the first period is still operating during the second period). Statistical tests to detect period and carryover effects are relatively insensitive, and analytic approaches to overcome these issues are generally unsatisfactory (Senn, 2002). However, these issues can be addressed with a separate analysis of first period data, which was possible in this meta-analysis. Finally, all trials involved one-off treatments and varying measures of side effects and had relatively short follow-up; hence, the safety and efficacy of long-term treatment remain uncertain.

Strengths of this review include the reporting of additional unpublished data from numerous trials and analyses of only the first treatment period, increasing the reliability. It is also the first review to compare results of low-dose and very low-dose ketamine, suggesting larger benefits with the former. The consistency of the findings described in this review suggest a true treatment effect in improving depressive symptoms, albeit temporarily for most. Several additional strands of evidence indicate that the benefit observed in these trials is a result of depression treatment, rather than nonspecific mood elevation or a ‘high’ following ketamine administration: there is improvement in core depressive symptoms such as sadness, suicidality, and helplessness; the time course of benefits is in the following days and sometimes weeks, whereas mood elevation with drugs of abuse is generally limited to the time of intoxication; the response pathway can be interrupted in animal models of depression treatment (Autry et al., 2011); and postoperative mood improvement occurs among patients given ketamine intra-operatively, who would have been unaware of immediate subjective effects (Kudoh et al., 2002; Argiriadou et al., 2004). Recent trials have also suggested possible benefits among patients with posttraumatic stress disorder (Whiteford et al., 2015) and in obsessive compulsive disorder (Rodriguez et al., 2013), and there is encouraging initial evidence comparing ketamine with ECT (Ghasemi et al., 2014).

### Relevance for Clinical Practice and Research

Most clinicians, drug regulators, and health funders will require further research evidence before low-dose ketamine is adopted in clinical practice. The results of this review have several implications for future research.

First, research is urgently required on efficacy and safety of longer treatment regimens. A key issue is whether the ketamine response can be maintained or even enhanced by repeated dosing, such as the 2 to 3 times weekly schedule that has been assessed in some case series with promising findings (Price et al., 2009; aan het Rot et al., 2010; DiazGranados et al., 2010b; Larkin and Beautrais, 2011; Rasmussen et al., 2013; Shiroma et al., 2014). Safety, tolerability, and abuse potential of repeated dosing is a critical issue for future trials. While some reassurance can be taken from the much more extensive use of i.v., oral, and sublingual ketamine in chronic pain control (Chong et al., 2009), future longer term trials must assess safety in this patient population. Trials should assess the likelihood of abuse potiential in subjects with major depression, while incorporating strategies to mitigate this.

Second, trials should adopt parallel designs rather than crossover designs, since benefits remain for more than 1 week in a significant proportion of patients; hence, this situation does not meet the fundamental criterion for crossover trials to have a stable disease baseline and short-lived treatment effect in all patients. Third, trials should recruit many times more patients, so that the effects in different subgroups can be assessed reliably given the considerable heterogeneity in response. Fourth, trial designs should reflect the possibility of different effects among patients with bipolar and unipolar disorder. Fifth, there is no evidence concerning first-line treatment of major depressive disorder: ketamine may have a role to play in patients with severe presenting symptoms, including suicidality, and could conceivably cover the lag phase before SSRI efficacy onset (and reduction of suicidal ideation may be particularly important in young adults). Further evidence of reduction in suicidality was recently provided by an additional trial published after our search cut-off date, conducted among 27 patients with mood and anxiety spectrum disorders who presented with clinically signiﬁcant suicidal ideation.(Murrough et al., 2015) Ketamine may also augment the response from standard treatments. For example, while ECT is indicated in severe depression with acute suicidality it may take a week or longer to alleviate symptoms and ketamine may play an important role by acting more quickly in this setting. Finally, further data on safety and efficacy of use in ‘real world’ clinical settings is clearly required, given that many of the trials were conducted in highly medically controlled environments. While i.v. infusions may be practical in service settings used for ECT, other formulations and delivery routes, such as intranasal spray, s.c., intramuscular, or oral, are likely to be more broadly applicable to clinical practice. Minimizing dissociative and hemodynamic side effects is important for widespread applicability, and this is likely to involve avoiding high peak plasma levels (Lai et al., 2014). However, very low doses are associated with lower efficacy, suggesting multiple low doses delivered gradually or sequentially may be optimal. Future trials could also assess ketamine enantiomers (Paul et al., 2009), metabolites (Zarate Jr et al., 2012), or other glutamatergic agents.

## Conclusions

This systematic review confirmed a large, rapid benefit in response and remission following a single dose of ketamine in patients with treatment resistant depression. There was also a reduction in suicidality. Transient psychotomimetic and heemodynamic effects occurred. There were no major medical events. Collectively, these data suggest ketamine has considerable promise for the acute treatment of major depression and provide the rationale for a large, parallel group, randomized, placebo-controlled trial to assess safety and efficacy of a longer course of ketamine in patients with severe mood disorders.

## Statement of Interest

Dr. Lapidus has received consulting fees from LCN consulting and serves on the advisory board for Halo Neuro Inc. In addition to performing ketamine research, he provides clinical treatments with ketamine. Until September 2014, Dr. Lapidus was at the Icahn School of Medicine at Mount Sinai, which has been named on a use patent on ketamine for the treatment of depression. The Icahn School of Medicine has entered into a licensing agreement for the use of ketamine as therapy for treatment-resistant depression and could potentially benefit if ketamine were to gain approval for the treatment of depression. Dr. Loo has received an honorarium from Lundbeck to speak at independent educational sessions organised by psychiatrists, for which Lundbeck provided funding. Dr. Glozier has received speaking honoraria from Lundbeck, Janssen, and Servier Laboratories, and advisory board fees from Lundbeck.

## Supplementary Material

supplementary Table 1
