# Supplementary material for: Effects of Low-Dose and Very Low-Dose Ketamine among Patients with Major Depression: a Systematic Review and Meta-Analysis
Source: Int J Neuropsychopharmacol. 2015 Nov 17;19(4):pyv124. doi: 10.1093/ijnp/pyv124 (PMC4851268; doi:10.1093/ijnp/pyv124)
Supplement: supplementary Table 1 [file S1_Table._Search_Strategies.docx]

| **Name of database** | **Search Keywords and Results** |
| --- | --- |
| **1.** **Medline** | 🞏 Search string using keywords   1. randomized controlled trial.pt. 2. controlled clinical trial.pt. 3. randomized.ab. 4. placebo.ab. 5. clinical trials as topic.sh. 6. randomly.ab. 7. trial.ti. 8. 1 or 2 or 3 or 4 or 5 or 6 or 7 9. exp Ketamine/ 10. Receptors, N-Methyl-D-Aspartate/ 11. 9 or 10 12. Depression/ 13. exp bipolar disorder/ or depressive disorder/ or depressive disorder, major/ or depressive disorder, treatment-resistant/ or dysthymic disorder/ 14. 12 or 13 15. 8 and 11 and 14 |
|  | 🞏 Result 70 publications (23/Jun/2014) *73 publications (16/Aug/2014)* |
| **2.** **Embase** | 🞏 Search string using keywords   1. 'controlled clinical trial'/exp 2. 'n methyl dextro aspartic acid receptor blocking agent'/exp 3. 'depression` /exp |
|  | 🞏 Result 167 publications (23/Jun/2014) *170 publications (16/Aug/2014)* |
| **3.** **PsycINFO** | 🞏 Search string using keywords   1. randomized controlled trial.mp. 2. controlled clinical trial.mp. 3. randomized.ab. 4. placebo.ab. 5. 1 or 2 or 3 or 4 6. ketamine/ 7. n-methyl-d-aspartate/ 8. 6 or 7 9. *"Depression (Emotion)"/ 10. *Major Depression/ or *Bipolar Disorder/ 11. *Treatment Resistant Depression/ 12. 9 or 10 or 11 13. 5 and 8 and 12 |
|  | 🞏 Result 33 publications (23/Jun/2014) *33 publications (16/Aug/2014)* |
|  | 🞏 Result 0 publications |
| **Total**  **(duplicates removed)** | Jun:215/270 (17 duplicates picked up by Endnote, 38 picked up manually)  Aug: 276 |

S1 Table. Search Strategies
